# Supplementary material for: Causal Relationship between Aging and Anorexia Nervosa: A White-Matter-Microstructure-Mediated Mendelian Randomization Analysis
Source: Biomedicines. 2024 Aug 16;12(8):1874. doi: 10.3390/biomedicines12081874 (PMC11351342; doi:10.3390/biomedicines12081874)
Supplement: Supplementary file 1 [file biomedicines-12-01874-s001.zip › Supplementary Figure/Figure S4/Singe SNP plot of MR analysis for TL to ubm-b-1776.pdf]

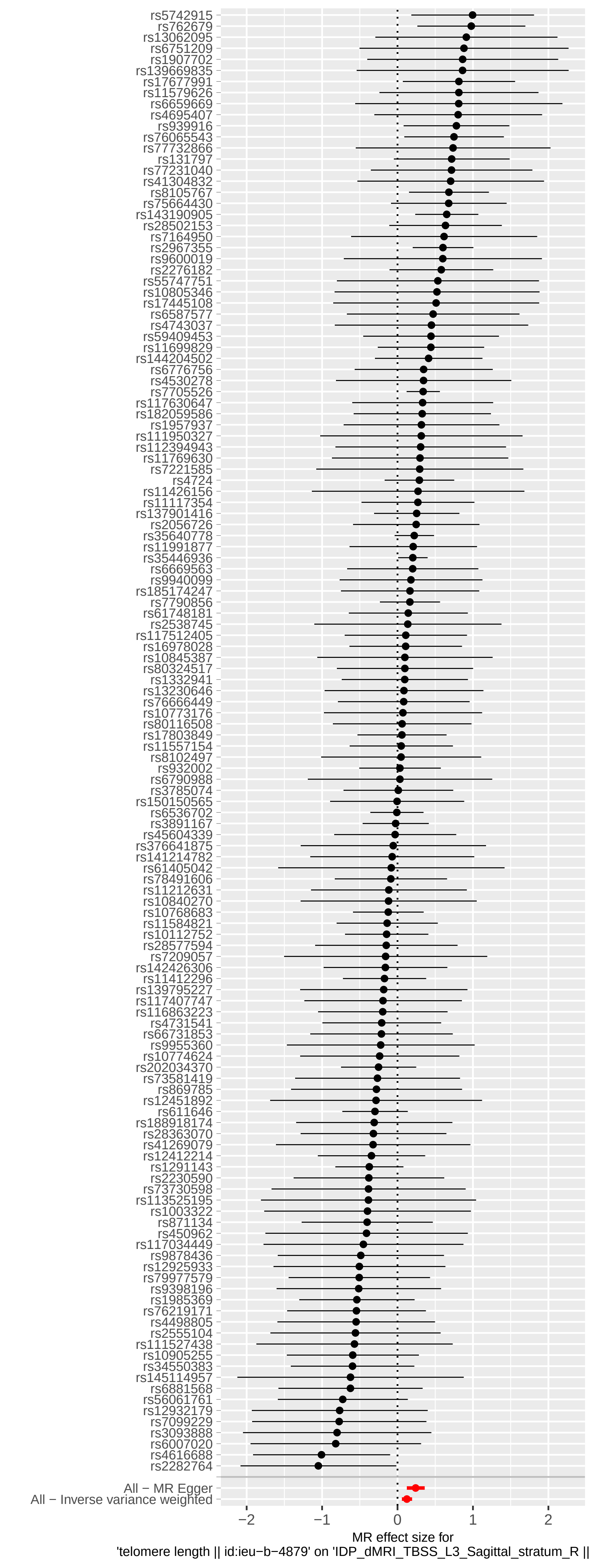

rs5742915
rs762679
rs13062095
rs6751209
rs1907702
rs139669835
rs17677991
rs11579626
rs6659669
rs4695407
rs939916
rs76065543
rs77732866
rs131797
rs77231040
rs41304832
rs8105767
rs75664430
rs143190905
rs28502153
rs7164950
rs2967355
rs9600019
rs2276182
rs55747751
rs10805346
rs17445108
rs6587577
rs4743037
rs59409453
rs11699829
rs144204502
rs6776756
rs4530278
rs7705526
rs117630647
rs182059586
rs1957937
rs111950327
rs112394943
rs11769630
rs7221585
rs4724
rs11426156
rs11117354
rs137901416
rs2056726
rs35640778
rs11991877
rs35446936
rs6669563
rs9940099
rs185174247
rs7790856
rs61748181
rs2538745
rs117512405
rs16978028
rs10845387
rs80324517
rs1332941
rs13230646
rs76666449
rs10773176
rs80116508
rs17803849
rs11557154
rs8102497
rs932002
rs6790988
rs3785074
rs150150565
rs6536702
rs3891167
rs45604339
rs376641875
rs141214782
rs61405042
rs78491606
rs11212631
rs10840270
rs10768683
rs11584821
rs10112752
rs28577594
rs7209057
rs142426306
rs11412296
rs139795227
rs117407747
rs116863223
rs4731541
rs66731853
rs9955360
rs10774624
rs202034370
rs73581419
rs869785
rs12451892
rs611646
rs188918174
rs28363070
rs41269079
rs12412214
rs1291143
rs2230590
rs73730598
rs113525195
rs1003322
rs871134
rs450962
rs117034449
rs9878436
rs12925933
rs79977579
rs9398196
rs1985369
rs76219171
rs4498805
rs2555104
rs111527438
rs10905255
rs34550383
rs145114957
rs6881568
rs56061761
rs12932179
rs7099229
rs3093888
rs6007020
rs4616688
rs2282764
All − MR Egger
All − Inverse variance weighted
−2
−1
0
1
2
MR effect size for
'telomere length || id:ieu−b−4879' on 'IDP_dMRI_TBSS_L3_Sagittal_stratum_R ||
